# Cerebrospinal fluid dysregulation and post-traumatic hydrocephalus after paediatric traumatic brain injury

**DOI:** 10.3389/fneur.2026.1878699

**Published:** 2026-07-29

**Authors:** Milan Makwana, Ajay Sinha

**Affiliations:** Department of Paediatric Neurosurgery, Alder Hey Children’s Hospital NHS Foundation Trust, Liverpool, United Kingdom

**Keywords:** cerebrospinal fluid dynamics, cerebrovascular autoregulation, intracranial compliance, paediatric traumatic brain injury, post-traumatic hydrocephalus

## Abstract

Post-traumatic hydrocephalus after paediatric traumatic brain injury is commonly framed as a delayed structural complication, yet the clinical literature indicates that this model is incomplete. Across paediatric neurocritical care and imaging studies, traumatic injury was associated with disturbed cerebrovascular autoregulation, impaired intracranial compliance, altered cerebral perfusion pressure targets, abnormal cerebrovascular reactivity, oxygenation abnormalities, and evolving glymphatic or perivascular changes. Normative paediatric studies likewise showed that cerebrospinal fluid physiology is developmentally dynamic, with age-dependent variation in aqueductal and spinal flow, glymphatic-associated indices, craniospinal compliance, and the relationship between brain and cerebrospinal fluid volumes. Together, these observations support the hypothesis that paediatric post-traumatic hydrocephalus may, in some patients, represent a downstream clinical expression of disturbed cerebrospinal fluid and intracranial fluid homeostasis, rather than a purely structural disorder of obstruction or absorption failure. The most direct clinical evidence came from infusion-study data in post-traumatic ventriculomegaly, in which resistance to cerebrospinal fluid outflow was higher in shunted cases, compliance was reduced, and structural imaging alone did not reliably distinguish probable hydrocephalus from ex vacuo ventricular enlargement. However, direct paediatric studies of incidence, physiology-based classification, and treatment selection remain limited. Current evidence supports consideration of a physiology-informed reframing of post-traumatic hydrocephalus, while also underscoring the need for longitudinal paediatric studies that link acute disturbances in cerebrospinal fluid dynamics with delayed ventriculomegaly, treatment response, and neurodevelopmental outcome.

## Introduction

Paediatric traumatic brain injury remains a major cause of death, disability, and long-term functional morbidity. Contemporary reviews consistently describe it as a leading cause of trauma-related death and disability in children and emphasise the long tail of neurocognitive, behavioural, educational, and quality-of-life sequelae amongst survivors (1–4). That framing is clinically important because the neurological burden after childhood traumatic brain injury is not exhausted by the acute management of intracranial hypertension. Rather, many clinically important complications unfold over time and reflect continuing physiological disturbance rather than isolated structural injury (2, 3).

Amongst those delayed complications, post-traumatic hydrocephalus occupies a difficult place in practise. It is clinically important because it may be treatable, but it is diagnostically challenging because ventricular enlargement after trauma can reflect true hydrocephalus, impaired compliance, altered pulsatility, cerebral atrophy, encephalomalacia, or combinations of these processes. In the only direct PTH-focused study, Lalou and colleagues described PTH as potentially underdiagnosed and under-treated, and showed that in post-traumatic ventriculomegaly, neither CT nor MRI descriptors alone consistently captured the dynamic abnormalities later demonstrated by infusion testing (5). That observation immediately complicates any paradigm that equates PTH with a static radiological appearance (Table 1).

**Table 1:** Selected clinical studies defining normal or developmentally varying paediatric CSF physiology.

Study	Design	Population	Physiological domain	Key finding	Evidence type	Directness to paediatric PTH
Sahoo et al. (2024) (6)	Cross-sectional real-time phase-contrast MRI flow study	Healthy volunteers including paediatric participants and adults	Ventricular, cervical and lumbar CSF flow; venous flow coupling	Paediatric participants showed age-dependent decreases in aqueductal, cervical and lumbar CSF net flow and pulsatility; adults did not show the same developmental change.	Normative paediatric/mixed-age physiological baseline	Normative paediatric baseline; indirect to PTH and used for age-calibrated interpretation of CSF dynamics.
Plata et al. (2022) (32)	Cine phase-contrast MRI study	Healthy children and adolescents	Aqueductal CSF flow	Reported paediatric aqueductal reference values for net flow, stroke volume and peak systolic/diastolic velocity; no clear age or sex effect was seen in this cohort.	Normative paediatric baseline	Normative paediatric baseline; indirect to PTH and useful as a reference framework, not trauma-specific evidence.
Demirtaş et al. (2020) (33)	Phase-contrast MRI study	Healthy children	Aqueductal CSF flow and anthropometric modifiers	Infants had lower cranial and net flow volumes; girls had lower net volume; lower head circumference was associated with lower flow volumes and aqueduct area.	Normative paediatric baseline	Normative paediatric baseline; indirect to PTH and supports age/head-size calibration of CSF flow interpretation.
Bateman and Brown (2012) (11)	Comparative MRI aqueduct-flow quantification study	Children with communicating hydrocephalus and controls	Aqueductal flow direction and magnitude	Controls showed an infantile inward-flow pattern and a mature outward-flow pattern, with an apparent transition around age 2 years.	Paediatric hydrocephalus physiology and normative developmental evidence	Indirect but paediatric; relevant to hydrocephalus physiology and developmental CSF dynamics, not post-traumatic PTH-specific.
Peterson et al. (2021) (9)	MRI volumetric cohort study	Healthy paediatric subjects across repeated MRI scans	Brain volume, CSF volume and brain-to-CSF ratio	Brain volume peaked at 10–12 years; the brain-to-CSF ratio followed a universal age-dependent relationship independent of sex or body size.	Normative paediatric volumetric baseline	Normative paediatric baseline; indirect to PTH and supports age-calibrated interpretation of ventriculomegaly and extra-axial CSF.
Wong et al. (2025) (7)	Normative DTI-ALPS imaging study	Children with normal MRI findings	Glymphatic-associated/perivascular transport	ALPS indices increased significantly with age and were bilaterally symmetrical.	Normative paediatric glymphatic baseline	Normative paediatric baseline; indirect to PTH and relevant to developmental interpretation of post-injury glymphatic/perivascular markers.
Ocamoto et al. (2024) (8)	Multicentre prospective observational non-invasive ICP waveform study	Healthy volunteers across the lifespan	Intracranial compliance waveform morphology	P2/P1 ratio and time-to-peak increased with age; men had lower values than women.	Mixed-age normative compliance baseline	Adult/mixed mechanistic baseline; indirect to paediatric PTH and limited by non-trauma, non-PTH design.
Rodriguez et al. (2022) (40)	Baseline compensatory reserve index study	Healthy participants including children	Compensatory reserve physiology	CRI varied with age, weight, estimated blood volume and heart rate.	Normative paediatric/mixed reserve physiology baseline	Normative paediatric baseline; indirect to PTH and relevant to age-dependent reserve physiology rather than hydrocephalus causation.

Directness refers to relevance to paediatric post-traumatic hydrocephalus, not study quality. Studies classified as indirect were used to support biological plausibility and hypothesis generation rather than to establish causation. ALPS, analysis along the perivascular space; CBF, cerebral blood flow; CPP, cerebral perfusion pressure; CRI, compensatory reserve index; CSF, cerebrospinal fluid; DTI, diffusion tensor imaging; ICP, intracranial pressure; MRI, magnetic resonance imaging; PTH, post-traumatic hydrocephalu.

The normal paediatric cerebrospinal fluid system is itself developmentally dynamic. Healthy-subject studies demonstrated age-related changes in aqueductal, cervical, and lumbar cerebrospinal fluid flow, developmental changes in glymphatic indices, age-dependent changes in intracranial compliance surrogates, and a universal age-dependent relationship between brain volume and cerebrospinal fluid volume across childhood (6–10). MRI-based work also suggested that aqueductal flow direction and magnitude differ between infancy and later childhood, with an apparent transition from an “infantile” to a “mature” flow pattern around 2 years of age (11). Taken together, these findings argue against treating the paediatric cerebrospinal fluid system as a scaled-down version of the adult system (Table 2).

**Table 2:** Selected clinical studies linking altered physiology after paediatric TBI to outcome or the post-traumatic hydrocephalus pathway.

Study	Design	Population	Physiological domain	Key finding	Evidence type	Directness to paediatric PTH
Agrawal et al. (2025)/STARSHIP (12)	Prospective multicentre validation monitoring study	Children with severe TBI	Cerebrovascular autoregulation/PRx	Pressure reactivity remained independently associated with 12-month outcome; lower PRx values were seen in children with more favourable outcomes.	Adjacent paediatric TBI physiology	Indirect; relevant to the proposed dysregulation pathway, but not direct PTH evidence.
Smith et al. (2025)/STARSHIP Part 2 (13)	Secondary PRx dose analysis	Children with severe TBI from the STARSHIP cohort	Magnitude and duration of pressure-reactivity disturbance	A greater cumulative burden of PRx disturbance, incorporating both magnitude and duration, was associated with worse 12-month outcome.	Adjacent paediatric TBI physiology	Indirect; supports time-dependent dysautoregulation as biological plausibility for later fluid dysregulation, not direct PTH evidence.
Bögli et al. (2025)/STARSHIP sub-analysis (15)	Pulse-shape/compliance monitoring sub-analysis	Children with severe TBI	Dynamic cerebral compliance/PSI	Higher PSI preceded ICP insults by a median of 8 min and predicted more severe subsequent insults.	Adjacent paediatric TBI compliance physiology	Indirect but highly relevant; supports early compliance deterioration as a plausible upstream pathway to later CSF decompensation.
Smith et al. (2025)/dynamic CPP analysis (14)	Secondary analysis of dynamic versus fixed CPP targets	Children with severe TBI from the STARSHIP cohort	CPP targets, lower limit of autoregulation and PRx-derived thresholds	Time spent below the lower limit of autoregulation was independently associated with worse outcome; the lower limit often exceeded 50 mmHg.	Adjacent paediatric TBI autoregulation/perfusion evidence	Indirect; relevant to proposed perfusion-autoregulation dysregulation pathway, not direct PTH evidence.
Kempen et al. (2026)/KidsBrainIT (80)	CPP dose–response modelling study	Paediatric TBI patients	CPP dose, ICP and cerebrovascular reactivity	Outcome modelling suggested a CPP corridor associated with better physiological conditions, approximately 56–89 mmHg when autoregulatory reactivity was preserved and ICP remained below 20 mmHg.	Adjacent paediatric TBI physiological modelling	Indirect; relevant to personalised perfusion/reserve management, not direct PTH evidence.
Hanalioglu/Appavu et al. (2023) (29)	Retrospective TCD study integrated with multimodality monitoring	Paediatric TBI patients undergoing TCD and multimodality monitoring	Cerebrovascular mechanics and CSF-space reserve	Reduced compliance of the CSF space, increased critical closing pressure and reduced diastolic closing margin were associated with poor outcome and/or increased ICP.	Adjacent paediatric TBI cerebrovascular/CSF-space reserve evidence	Indirect but mechanistically relevant; supports CSF-space reserve impairment after paediatric TBI, not direct PTH evidence.
Wolf et al. (2021) (42)	Proof-of-concept dynamic intracranial compliance study	Children with severe TBI	Dynamic intracranial compliance	Compliance derangement appeared to persist for several days after injury despite limited time with ICP > 20 mmHg.	Adjacent paediatric TBI compliance evidence	Indirect; supports persistent reserve derangement after injury, not direct PTH evidence.
Brady et al. (2009) (18)	Prospective observational continuous PRx monitoring study	Children with severe TBI	Cerebrovascular pressure reactivity	Preserved pressure reactivity was associated with survival.	Adjacent paediatric TBI autoregulation evidence	Indirect; foundational paediatric TBI autoregulation evidence relevant to the proposed pathway, not direct PTH evidence.
Adelson et al. (1997) (46)	Observational XeCT CBF and CO2 reactivity study	Infants and young children with severe TBI	Cerebral blood flow and CO2 vasoreactivity	Early low CBF and low CO2 vasoreactivity were associated with poor outcome, especially in the youngest children.	Adjacent paediatric TBI perfusion physiology	Indirect but paediatric; supports early age-sensitive perfusion failure as upstream biological plausibility, not direct PTH evidence.
van der Horn et al. (2025) (20)	Longitudinal paediatric mild TBI MRI study	Paediatric mild TBI patients assessed acutely and at follow-up	Cerebrovascular reactivity and pCASL perfusion	CVR abnormalities and cerebellar hypoperfusion persisted up to 4 months after injury.	Adjacent paediatric mild TBI cerebrovascular physiology	Indirect; relevant to persistence of post-traumatic fluid/vascular dysregulation, not direct PTH evidence.
An et al. (2025) (24)	DTI-ALPS imaging study in TBI with diffuse axonal injury	TBI patients including cases with DAI	Glymphatic-associated/perivascular transport	DAI was associated with lower ALPS indices, with worse values at higher DAI grade.	Mixed/adjacent TBI glymphatic imaging evidence	Indirect mechanistic evidence; supports disrupted clearance after TBI, not direct paediatric PTH evidence.
Lalou et al. (2020) (5)	Retrospective observational CSF infusion study	Patients with non-acute post-traumatic ventriculomegaly	CSF outflow resistance, pulse amplitude, compliance and ventricular enlargement	In post-traumatic ventriculomegaly, cases selected for shunting had higher Rout, with reduced compliance and inconsistent structural imaging descriptors.	Direct post-traumatic ventriculomegaly evidence	Direct to post-traumatic ventriculomegaly/PTH pathway; limited paediatric specificity.

Directness refers to relevance to paediatric post-traumatic hydrocephalus, not study quality. Studies classified as indirect were used to support biological plausibility and hypothesis generation rather than to establish causation. ALPS, analysis along the perivascular space; CBF, cerebral blood flow; Ci, compliance of the cerebrospinal space; CPP, cerebral perfusion pressure; CrCP, critical closing pressure; CVR, cerebrovascular reactivity; DAI, diffuse axonal injury; DCM, diastolic closing margin; ICP, intracranial pressure; LLA, lower limit of autoregulation; MMM, multimodality monitoring; pCASL, pseudo-continuous arterial spin labelling; PRx, pressure reactivity index; PSI, pulse-shape index; PTH, post-traumatic hydrocephalus; Rout, resistance to CSF outflow; TBI, traumatic brain injury; TCD, transcranial Doppler; XeCT, xenon-enhance.

This developmental context matters after injury because the same traumatic brain injury may perturb several coupled fluid systems at once: cerebral blood flow, venous outflow, intracranial compliance, pulse transmission, perivascular exchange, and cerebrospinal fluid circulation. The paediatric traumatic brain injury monitoring literature repeatedly showed that disturbed autoregulation, impaired cerebral perfusion pressure control, altered cerebrovascular reactivity, and reduced compliance are associated with worse outcomes (12–19). Mild traumatic brain injury studies, meanwhile, demonstrated persistent abnormalities in cerebral blood flow, cerebrovascular reactivity, and glymphatic or perivascular imaging markers well beyond the immediate post-injury period (20–25). These data suggest that fluid dysregulation begins early and may precede overt ventriculomegaly (Table 3).

**Table 3:** Evidence map for the proposed PTH pathway.

Proposed pathway component	Direct paediatric PTH evidence	Indirect paediatric evidence	Adult/mixed mechanistic evidence	Strength of inference
Ventriculomegaly versus ex vacuo enlargement	Limited	Moderate	Moderate	Moderate
Raised CSF outflow resistance	Limited	Limited	Moderate	Low–moderate
Reduced compliance	Limited	Moderate	Moderate	Moderate
Autoregulatory disturbance	None direct for PTH	Strong in paediatric TBI	Moderate	Indirect but biologically plausible
Glymphatic/perivascular dysfunction	None direct for PTH	Emerging	Moderate	Low
Treatment selection by physiology	Very limited	Limited	Moderate	Hypothesis-generating

A strictly dichotomous model of cerebrospinal fluid physiology based only on obstruction versus absorption failure, therefore, seems too narrow for paediatric trauma. Normative imaging studies highlight pulsatility, venous coupling, perivascular transport, and craniospinal compliance; trauma studies highlight failure of autoregulatory control, disturbed compliance, perfusion mismatch, and altered fluid exchange; and the post-traumatic hydrocephalus study highlights the inadequacy of imaging alone in post-traumatic ventriculomegaly (5, 6, 12, 15, 26–29). A more clinically useful framework is to view post-traumatic hydrocephalus as one end of a spectrum of disturbed cerebrospinal fluid regulation, in which altered perfusion, compliance, pulsatility, and outflow interact over time. This narrative review synthesises direct and indirect clinical and imaging evidence relevant to paediatric CSF physiology, paediatric TBI pathophysiology, and post-traumatic ventriculomegaly. Its aim is to evaluate a hypothesis-generating framework in which paediatric PTH is considered not only as a structural complication, but also as a possible downstream expression of disturbed fluid regulation, while explicitly identifying where evidence is direct, indirect, or extrapolated (Table 4).

**Table 4:** Evidence classification.

Evidence class	Definition	Examples in manuscript
Direct paediatric PTH evidence	Paediatric or mixed post-traumatic ventriculomegaly/PTH studies assessing diagnosis, physiology, or treatment	Lalou et al. infusion study
Adjacent paediatric TBI physiology	Paediatric TBI studies of autoregulation, perfusion, compliance, oxygenation, ICP, outcome	STARSHIP, Appavu, Wolf, Vavilala
Normative paediatric CSF physiology	Healthy paediatric CSF flow, volume, compliance, glymphatic or ONSD reference studies	Sahoo, Peterson, Wong, Bateman and Brown
Adult/mixed mechanistic evidence	Adult or mixed studies used only for biological plausibility	Han, Xu, Gruszecki, adult ALPS/perivascular studies
Conceptual inference	Interpretive synthesis not directly proven by longitudinal paediatric PTH cohorts	Proposed physiology-informed pathway

### Search strategy and evidence classification

We searched PubMed/MEDLINE, Embase, Scopus/Web of Science, and Google Scholar for studies relating to paediatric traumatic brain injury, post-traumatic hydrocephalus, ventriculomegaly, cerebrospinal fluid dynamics, autoregulation, compliance, perfusion, glymphatic imaging, and paediatric normative CSF physiology. Searches were performed from January 1996 to January 2026.

Search terms included combinations of: “paediatric” OR “pediatric” OR “child*” OR “infant”; “traumatic brain injury” OR “head injury” OR “concussion”; “post-traumatic hydrocephalus” OR “ventriculomegaly” OR “hydrocephalus”; “cerebrospinal fluid” OR “CSF dynamics” OR “CSF flow”; “autoregulation” OR “pressure reactivity” OR “cerebral perfusion pressure”; “intracranial compliance” OR “pulse amplitude”; “glymphatic” OR “perivascular space.”

Studies were included if they addressed one or more of the following: direct post-traumatic hydrocephalus or post-traumatic ventriculomegaly; paediatric traumatic brain injury physiology; normative paediatric CSF physiology; paediatric hydrocephalus flow/compliance physiology; or adult/mixed-population mechanistic studies relevant to CSF dynamics where paediatric evidence was unavailable.

Studies were excluded if they were unrelated adult hydrocephalus studies without mechanistic relevance to trauma or CSF physiology, isolated case reports without physiological or diagnostic relevance, or articles without accessible full text.

## Normal cerebrospinal fluid physiology in children

### Cerebrospinal fluid production and secretion

The normal paediatric physiology literature is much richer in imaging and volumetric studies than in direct secretion studies, so any account of production must remain indirect. That limitation is important because many downstream arguments about paediatric hydrocephalus still assume stable production against variable outflow, whereas the literature reviewed here is more focused on the behaviour of the intracranial fluid system as a whole (11, 30).

The literature, however, provides a developmental volumetric context for structures implicated in CSF production and intracranial fluid balance. In a paediatric CT study of 229 scans from birth to 10 years, Hashimoto and colleagues found that choroid plexus volume and lateral ventricular volume both increased rapidly until approximately 1 year of age, after which choroid plexus volume plateaued around 1.5 mL and lateral ventricular volume around 10 mL (31). Although this study was undertaken in the context of choroid plexus hyperplasia and hydrocephalus rather than trauma, it provides clinically relevant developmental baseline information: structures often invoked in discussions of “CSF overproduction” are themselves changing quickly in infancy, which cautions against interpreting ventricular or plexus size without age calibration (31).

A complementary MRI-based study by Peterson and colleagues quantified brain and CSF volumes in 505 healthy paediatric subjects from birth to 18 years across 1,067 scans. Brain volume peaked at 10–12 years, males had larger age-adjusted absolute brain volumes than females, but the ratio of brain to CSF volume followed a universal age-dependent pattern independent of sex or body size (9). For clinicians, that finding is particularly valuable because it suggests that the developmental relationship between parenchyma and CSF, rather than either compartment in isolation, may be the most stable reference frame for interpreting abnormal ventricular or extra-axial fluid accumulation in children (9).

**Figure 1:**
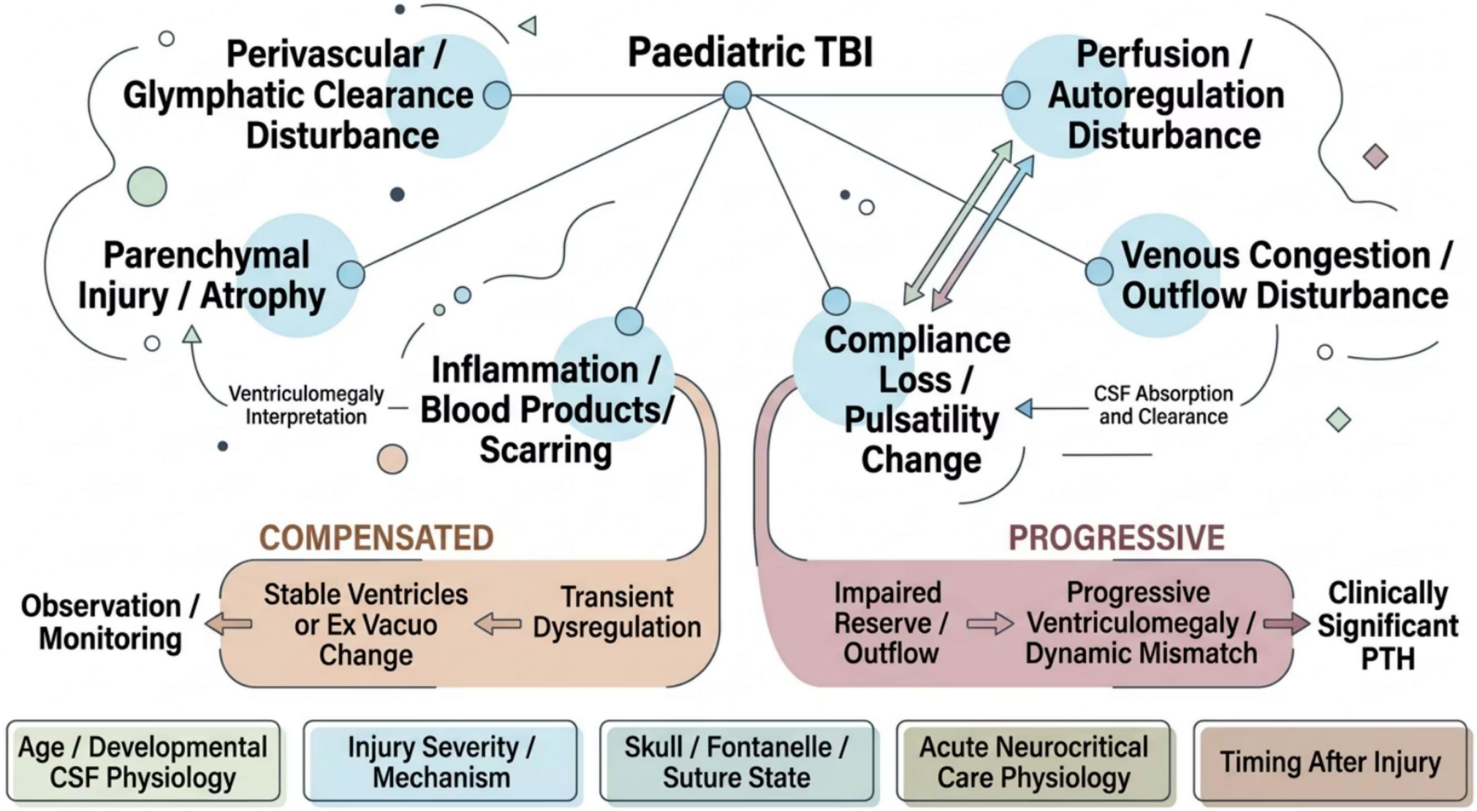
Hypothesis-generating model of paediatric post-traumatic hydrocephalus as a potential downstream manifestation of interacting disturbances in perfusion, autoregulation, venous outflow, compliance, pulsatility, parenchymal injury, and CSF clearance. The model distinguishes compensated trajectories—including stable ventriculomegaly or ex vacuo enlargement—from progressive trajectories in which impaired reserve and outflow may culminate in clinically significant PTH. Developmental CSF physiology, age, injury mechanism, and timing after trauma are proposed modifiers. The model is not a validated clinical decision pathway.

These studies do not directly measure secretion, but together they indicate that childhood cerebrospinal fluid physiology must be interpreted within a moving developmental architecture. In infancy and early childhood, the size of the choroid plexus, ventricles, brain, and extra-axial spaces is changing rapidly, and the relative proportions of these compartments are more informative than isolated measurements alone (9, 31). This matters for post-traumatic hydrocephalus because apparent “excess CSF” may reflect abnormal scaling between compartments rather than a simple increment in ventricular volume. The paediatric hydrocephalus flow study by Bateman and Brown also implies that the same ventricular compartment may participate in different net flow states at different developmental stages (11). That observation weakens any assumption that secretion and absorption can be inferred from ventricular size or aqueductal patency alone. At a minimum, paediatric cerebrospinal fluid production should be understood as operating within a developmental hydraulic network whose apparent behaviour change with age and disease state (Figure 1).

### Cerebrospinal fluid circulation pathways

The clearest clinical data on paediatric CSF circulation come from phase-contrast MRI. Plata and colleagues evaluated 32 healthy children and adolescents with cine phase-contrast MRI and reported mean aqueductal values for net flow, stroke volume, mean velocity, peak systolic velocity, and peak diastolic velocity (32). In that cohort, basic aqueductal flow parameters were not significantly affected by age, sex, or body surface area (32). On first reading, these findings suggest a degree of stability in paediatric aqueductal flow across later childhood.

However, other paediatric imaging studies show that the picture is more nuanced. In a larger prospective study of 137 healthy participants aged 2 to 204 months, Demirtas and colleagues found that peak velocity was higher in children than in infants, cranial and net volumes were lower in infants, girls had lower net volume than boys, and children with lower head circumference had lower forward, reverse, and net flow volumes together with smaller aqueductal diameter (33). These results do not entirely align with the smaller series by Plata et al., but that discrepancy is itself clinically informative: paediatric aqueductal flow metrics show substantial methodological and biological variability, and age-related effects may depend on which component of flow is measured and over what developmental span (32, 33).

Real-time phase-contrast work by Sahoo and colleagues adds an important spinal dimension. In 44 healthy volunteers aged 5–40 years, they quantified CSF flow at the aqueduct, cervical spine, and lumbar spine, and venous flow in epidural, jugular, and caval compartments (6). In adults, flow parameters did not change significantly, whereas paediatric subjects showed significant age-dependent decreases in CSF net flow and pulsatility at all three CSF levels (6). This study is especially valuable because it places ventricular and spinal CSF dynamics within a single framework and shows that age dependence is not confined to the aqueduct.

The most conceptually provocative paediatric circulation study is the magnetic resonance aqueduct flow study by Bateman and Brown in normal and hydrocephalic children. They identified two distinct control patterns: an infantile pattern with net flow directed into the ventricles, and a mature pattern with net flow directed out of the ventricles, with an apparent transition around 2 years of age (11). In infants with communicating hydrocephalus, the direction changed, but the magnitude remained similar to controls, whereas in older hydrocephalic children, net aqueductal flow was elevated sevenfold with preserved direction (11). These observations are difficult to reconcile with overly linear models of one-way cerebrospinal fluid bulk flow and support the idea that net aqueductal flux reflects broader developmental and hydraulic context rather than a single passive stream (11).

Earlier work reinforces both the promise and the limitations of aqueductal flow imaging. In children with normal and dilated ventricles, phase-contrast MRI demonstrated marked inter-individual variation and overlap in aqueductal velocities and flow rates; measurable flow could be present in shunted patients, and aqueductal stenosis could present with either pronounced or absent flow (34). Similarly, Hofmann and colleagues reported substantial interindividual variability in healthy controls and concluded that single-parameter flow measurements are limited in sensitivity and specificity for identifying disturbed CSF dynamics at the individual level (35). These findings are highly relevant when considering suspected PTH: flow imaging can reveal physiology, but normal-range overlap is substantial (34, 35).

### Cerebrospinal fluid absorption and clearance

Direct paediatric clinical evidence regarding arachnoid granulations or spinal nerve root absorption pathways was not specified. Instead, the absorption and clearance literature is dominated by studies of perivascular or glymphatic transport, pulsatility, and coupled venous–cerebrospinal fluid dynamics. This reflects a broader shift in the field from static outflow models toward distributed clearance concepts (6, 7, 26).

In normative paediatric imaging, Wong and colleagues used DTI-ALPS in 72 children with normal MRI and showed a significant age-related increase in ALPS indices, with no significant left–right difference (7). In practical terms, this suggests that glymphatic-associated signal characteristics are developmentally regulated in childhood and cannot be treated as age invariant (7). That matters when interpreting post-injury ALPS studies, because “abnormal” values may depend partly on where a child sits on a developmental trajectory.

Human data supporting perivascular transport as a dynamic physiological process were provided by Han and colleagues, who used dynamic diffusion tensor imaging to study perivascular CSF flow during the cardiac cycle. Although their cohort was adult, they showed that perivascular CSF diffusion properties were aligned with neighbouring arterial flow and changed with vasodilation, supporting arterial pulsation as a driver of perivascular flow (26). This is not a paediatric study, but it is relevant because it identifies a mechanism by which vascular and CSF compartments are mechanically coupled.

Other human studies suggest that clearance-related physiology is also influenced by movement and low-frequency oscillation. Xu and colleagues showed that head-nodding altered cerebrospinal fluid flow at the cranio-cervical junction and increased lumbar cerebrospinal fluid pressure in healthy volunteers (28). Gruszecki and colleagues demonstrated oscillations in human subarachnoid space width across a broad frequency range and relationships between these oscillations, blood pressure, and age (27). Although indirect, these data support the idea that clearance cannot be reduced to a constant passive sink; it is an oscillatory, mechanically modulated process involving vascular, dural, and subarachnoid structures (26–28).

For paediatric neurosurgery, the main implication is that “absorption failure” may be a downstream summary label rather than a mechanistic diagnosis. The clinical literature shows that clearance-related physiology is developmentally variable, pulsatile, linked to arterial and venous dynamics, and can be measured only imperfectly with current imaging. That is a more complex starting point for understanding post-traumatic hydrocephalus than a single-point blockage in a bulk-flow circuit (6, 7, 26, 27).

### Pressure–volume relationships and developmental intracranial compliance

The continuity between intracranial and perioptic subarachnoid spaces is also relevant to normal pressure transmission. Although the most detailed discussion comes from a review aimed at paediatric radiologists, which emphasised that the perioptic space is the optic nerve subarachnoid space in continuity with the intracranial SAS, and that variation in its calibre can reflect intracranial pressure states (36). Normative paediatric CT and MRI studies indirectly support that claim by showing that ONSD and related measures change with age and, in some series, sex, even in children without intracranial pathology (37–39). This matters because any trauma study using ONSD as a bedside surrogate is only interpretable against these age-sensitive anatomical baselines.

Several studies show that the pressure–volume environment of the child is also developmentally dynamic. Using a non-invasive waveform-derived method, Ocamoto and colleagues studied 188 healthy volunteers and found that the P2/P1 ratio and time-to-peak rose with age, while men had lower values than women (8). The cohort extended beyond childhood, but the data support age-dependent effects on compliance-related waveform morphology, which is important when extrapolating thresholds across infants, older children, and adults (8).

Rodriguez and colleagues similarly reported age-related variability in the compensatory reserve index (CRI) in a healthy paediatric cohort, with strong correlations between CRI and age, weight, estimated blood volume, and heart rate (40). Although CRI is not a direct CSF metric, it reflects systemic compensatory reserve and reinforces the principle that haemodynamic and intracranial reserve variables are not age neutral in children (40).

MRI-based modelling by Burman and colleagues extended this by estimating patient-specific distributions of craniospinal compliance across a wide age range. Their work suggested that compliance distribution between cranial and spinal compartments can be derived non-invasively from vascular and cervical cerebrospinal fluid flow measurements and that this distribution is related to intracranial pressure (41). This study is an attractive concept because it shifts attention from total compliance to where compliance resides. That distinction may be particularly relevant in paediatric trauma, where skull properties, open sutures or fontanelles, and spinal buffering capacity may differ substantially across ages.

In infancy, fontanelle-based work provides further developmental nuance. Massager and colleagues recorded anterior fontanelle pressure in healthy infants and showed that mean pressure, pulse amplitude, and pressure-wave characteristics varied with post-conceptional age in a sigmoidal pattern, with low-amplitude plateau-like waves present in many infants beyond 49 post-conceptional weeks (10). These observations suggest that apparently abnormal wave phenomena can be physiological at certain developmental stages and that age calibration is essential when interpreting infant pressure dynamics (10).

Taken together, the normal paediatric literature supports four conclusions: first, ventricular and spinal cerebrospinal fluid flow vary with age; second, aqueductal flow direction itself may change during early childhood; third, glymphatic-associated indices mature across childhood; and fourth, compliance-related measures are age dependent (6–8, 10, 11, 32, 33, 40). Any post-traumatic framework that ignores those developmental baselines risks confusing normal maturation, compensated abnormality, and true hydrocephalic failure.

### Modern concepts in cerebrospinal fluid dynamics beyond bulk flow

The older bulk-flow model imagines CSF as being produced upstream, passing unidirectionally through the ventricles and subarachnoid spaces, and then being absorbed downstream. The reviewed literature does not support such a simple picture. Instead, it presents CSF dynamics as an interaction amongst pulsatility, venous coupling, perivascular transport, compliance, and regional oscillation (6, 11, 26, 27, 30).

One line of evidence comes from arterial pulsation. Han and colleagues showed that dynamic diffusion measures within perivascular CSF change with the cardiac cycle and with vasomotor state, consistent with arterial pulsation accelerating perivascular flow in the human brain (26). Although the cohort was not paediatric, this mechanism is highly relevant to trauma because vascular dysregulation is ubiquitous after TBI, making it plausible that injured brains experience clearance disturbance even before ventricular enlargement develops (26).

A second line of evidence comes from developmental glymphatic imaging. In normative children, ALPS indices increased with age, implying maturation of glymphatic-associated transport across childhood (7). When set beside trauma studies showing altered ALPS after brain injury, these data suggest that post-traumatic glymphatic changes must be interpreted relative to developmental state, not against a single “normal” paediatric value (7, 24, 25).

A third line of evidence concerns venous-CSF coupling. Sahoo et al. demonstrated age-dependent changes in both CSF and venous flow from the aqueduct to the lumbar spine, implying that intracranial fluid regulation cannot be separated from venous drainage (6). Low-frequency oscillation studies of subarachnoid space width similarly linked SAS dynamics to blood pressure and age (27). These studies do not describe hydrocephalus directly, but they show that CSF behaviour is inseparable from vascular mechanics.

Movement-related modulation offers a fourth perspective. Xu et al. reported that head nodding altered cranio-cervical CSF flow and increased lumbar pressure, suggesting that extra-neural mechanical factors can affect CSF dynamics *in vivo* (28). This is a striking reminder that the CSF system is not driven only by secretion and absorption; it is also shaped by posture, motion, vascular pulsation, and biomechanical transmission across the cranio-cervical junction (28).

The paediatric hydrocephalus study by Bateman and Brown further undermines the bulk-flow model. Their finding of inward aqueductal net flow in infancy and outward net flow in later childhood, together with markedly increased flow magnitude in older children with communicating hydrocephalus, suggests that measured aqueductal flux is better understood as a marker of system state than as direct evidence of a single bulk stream (11). In other words, the direction and magnitude of aqueductal flow reflect how the wider system buffers, pulsates, and exchanges fluid.

These modern concepts are clinically useful in trauma because they help explain why fluid dysregulation may be present despite apparently modest ventricular enlargement. If compliance is falling, pulsatility is changing, cerebrovascular reactivity is impaired, and perivascular clearance is abnormal, then the child may already be moving toward symptomatic CSF dysregulation before formal radiological hydrocephalus is obvious (15, 20, 24, 29, 42). This is precisely the sort of temporal disconnect that a static “obstruction versus absorption” framework would miss.

## Traumatic brain injury in children: pathophysiological context

Paediatric TBI is physiologically heterogeneous, encompassing severe accidental injury, mild concussion, sports-related repetitive head impacts, and inflicted traumatic brain injury in infants and young children (12, 20, 22, 43–46). That heterogeneity matters because the same final outcome label, such as ventriculomegaly or poor neurological recovery, may emerge from very different patterns of perfusion failure, compliance loss, vascular dysregulation, and tissue injury.

In severe TBI, several older paediatric studies described early cerebral blood flow depression followed by variable hyperaemia. This pattern was not merely descriptive; it intersected with age, CO2 reactivity, and outcome. In infants and young children with severe TBI, Adelson and colleagues reported mean admission CBF around 25 mL/100 g/min, with 77% of early studies showing values ≤20 mL/100 g/min; by 24 h and up to 6 days, mean CBF increased significantly, and CBF ≤ 20 mL/100 g/min at any time was associated with poor outcome (46). A later larger series similarly found low admission CBF, increasing CBF by post-injury days 1–2, and particularly poor outcomes in children younger than 2 years and in those with low early CBF (47). These studies suggest that post-traumatic cerebral perfusion in children is dynamic, developmentally contingent, and mechanistically linked to vasoregulatory reserve rather than to blood pressure alone. These data suggest that paediatric TBI often begins with a phase of hypoperfusion rather than simple post-traumatic hyperaemia.

Yet early perfusion is not uniform. In 42 children with severe TBI and CPP > 40 mmHg, Vavilala and colleagues still observed low flow velocity in some patients and high flow velocity in others, with most remaining in a normal range and only low haematocrit associated with high flow velocity (48). The implication is that a threshold CPP does not guarantee physiological adequacy. That theme persists across more modern autoregulation work, which shows that the effect of pressure on cerebral blood flow depends on the state of autoregulatory control rather than on mean pressure alone (12, 14, 49).

Oedema and delayed intracranial hypertension form another part of the pathophysiological context.

In practical terms, this means that a child may deteriorate after an initially acceptable pressure course. In a retrospective paediatric series, delayed intracranial hypertension occurred in 13% of severe TBI cases and was associated with oedema on the initial CT scan (50). Earlier adult or mixed-age work linked raised ICP to increased cerebral blood volume rather than a simple linear relation with cerebral blood flow, supporting the concept that swelling can reflect vascular volume dysregulation as much as water accumulation (51). That distinction is highly relevant to later hydrocephalus, because vascular engorgement, impaired venous return, interstitial fluid accumulation, and reduced CSF reserve all compete within the same fixed cranial compartment. These observations are relevant to PTH because they suggest that traumatic fluid imbalance begins in the vasculature and interstitium long before hydrocephalus is formally diagnosed.

Studies also show that metabolic disturbance is central. In non-invasive paediatric studies, severe TBI was associated with predominant hypoperfusion and low oxygen metabolic index, supporting metabolic dysfunction (52). Prospective monitoring studies demonstrated that reduced brain tissue oxygenation occurs early after severe paediatric TBI, that cumulative burdens of low PbtO2 are greater than single time-point prevalence suggests, and that PbtO2 is related to CPP and ICP rather than existing as an isolated variable (53–56). Adult and mixed-age work shows that metabolic failure, reflected by elevated lactate/pyruvate ratios, tracks with adverse physiology and poor outcome (57, 58).

Cerebrovascular autoregulation is a further central determinant of secondary injury. Early paediatric studies showed that impaired autoregulation was associated with poor outcomes after severe TBI and that younger children, especially those with inflicted injury, frequently had abnormal autoregulatory indices (19, 45, 46). Prospective PRx work then demonstrated a strong association between preserved pressure reactivity and survival (18). More recently, the STARSHIP programme validated the association between PRx derangement and 12-month outcome in a multicentre prospective UK cohort, extending older single-centre findings into a contemporary paediatric dataset (12, 13).

The pressure–volume environment is also altered after injury. Wolf et al. showed that a novel pressure–PCO2 compliance index could be derived from routine monitoring and suggested prolonged compliance derangement for up to 5 days after severe paediatric TBI, despite relatively modest time spent with ICP above 20 mmHg (42). Appavu and colleagues found that reduced compliance of the cerebrospinal space, increased critical closing pressure, and reduced diastolic closing margin were associated with poor outcome and, in some cases, with higher ICP (29). Taken together, these studies imply that intracranial fluid buffering may be substantially abnormal even when the mean ICP alone looks only intermittently concerning.

This is the pathophysiological setting in which post-traumatic hydrocephalus must be understood. In paediatric TBI, the brain experiences evolving hypoperfusion or hyperperfusion, oxygenation deficits, autoregulatory failure, impaired compliance, altered pressure transmission, and likely disrupted perivascular exchange (18, 19, 29, 42, 46, 47, 52, 53). These findings make it plausible that, in some children, PTH may emerge from unresolved disturbances in perfusion, compliance, pulsatility, and CSF outflow rather than from a single isolated structural mechanism. It is more plausibly the delayed structural expression of these earlier dynamic disturbances.

## Alterations in cerebrospinal fluid dynamics after paediatric traumatic brain injury

### Acute changes

The acute period after paediatric traumatic brain injury is characterised by marked disturbance of perfusion and pressure regulation. Historical physiological studies also show that this disturbance is sensitive to therapy. Low early CBF was prominent in both the 1997 infant and young child series by Adelson et al. and the larger 2011 cohort by Figaji et al., with poor outcome concentrated amongst those with very low early flow (46, 47). Because these studies span infancy to adolescence, they also suggest that age modulates the physiological response to trauma, with the youngest patients appearing particularly vulnerable to early hypoperfusion (46, 47). Hyperventilation could reduce regional cerebral blood flow in severely head-injured children, highlighting the possibility that standard intracranial pressure–directed manoeuvres may worsen perfusion in vulnerable patients (59). Pressure autoregulation testing and vasomotor reactivity studies from the same era similarly linked blood pressure and flow velocity to outcome, suggesting against the use of uniform protocols (60, 61). These older studies are methodologically heterogeneous, but they are strikingly consistent with more recent calls for personalised physiology rather than one-size-fits-all targets.

The acute haemodynamic state is not captured fully by CPP thresholds alone. Vavilala and colleagues found that low, normal, and high middle cerebral artery flow velocities were observed in severe paediatric TBI, despite a CPP > 40 mmHg (48). Earlier retrospective outcome work also showed that no child with a mean CPP < 40 mmHg survived, but that above this floor, outcome did not improve in a simple linear fashion across higher CPP bands (62). These studies imply that pressure targets are necessary but insufficient; the key question is whether blood flow and vascular reactivity remain physiologically matched to that pressure.

Autoregulatory studies make this mismatch explicit. In 21 children with severe TBI, PRx values consistent with preserved cerebrovascular reactivity were strongly associated with survival (18). In a separate cohort of 36 children, impaired autoregulation and hypotension were independent predictors of poor 6-month outcome (19). These observations have now been strengthened by multicentre work: the STARSHIP validation study showed that PRx remained associated with the 12-month outcome after adjustment for clinical predictors, and STARSHIP Part 2 showed that both the magnitude and the duration of PRx disturbance carry prognostic information (12, 13). Thus, the acute post-traumatic problem is not simply “raised ICP,” but a broader failure of blood pressure-to-flow buffering.

Compliance derangement is another acute CSF-relevant abnormality. Older waveform studies support the same principle from a different angle. Slow pressure waves of possible autonomic origin have been described in severely head-injured children, while broader critical care work emphasised that ICP waveform morphology, pulse amplitude, and compensatory reserve may all carry information beyond mean ICP (63, 64). In the STARSHIP compliance sub-analysis, higher pulse shape index values preceded ICP insults by a median of 8 min, and higher pre-insult compliance abnormality was associated with greater insult magnitude and longer insult duration (15). In the proof-of-concept compliance study by Wolf et al., the derived PCI was frequently abnormal even when the proportion of time with ICP > 20 mmHg was comparatively low (42). The PVI literature in mixed-age brain-injured cohorts also linked pressure–volume relations to autoregulation status (65). Although these studies do not define paediatric PTH directly, they reinforce the idea that acute injury alters the shape and adaptability of the intracranial fluid system, rather than only its average pressure.

Older work on intracranial compliance and ICP waveform behaviour similarly suggested that bedside compliance measures capture information not conveyed by mean pressure alone (66, 67). For a review focused on CSF dynamics, this is critical: pressure–volume reserve is itself a CSF-related physiological variable, not merely a consequence of lesion burden.

Acute oxygenation and metabolism studies are consistent with this interpretation. Brain tissue oxygen monitoring demonstrated that low PbtO2 events are common early after paediatric TBI when measured cumulatively, and low CPP or elevated ICP are associated with worse oxygenation metrics (53–56). Figaji et al. further reported that hypoperfusion and low oxygen metabolic index predominate after severe paediatric TBI (52). These abnormalities likely reflect combined failures of flow, diffusion, and cellular metabolism, but, from a systems perspective, they also imply inefficient coupling amongst the vascular, interstitial, and CSF compartments.

### Subacute and chronic changes

CSF-related dysregulation often persists well beyond the initial resuscitation period. In paediatric mild TBI, van der Horn et al. reported persistent cerebrovascular reactivity abnormalities up to 4 months after injury, with increased response amplitude in some regions, delayed latency in others, and cerebellar hypoperfusion on pCASL (20). Barlow et al. found that children with persistent post-concussion symptoms had higher cerebral blood flow than those with good recovery and that the trajectory of CBF decline over time predicted clinical recovery (21). Together, these studies indicate that the post-traumatic fluid environment remains dynamic even when overt intracranial hypertension is absent.

Sports-related concussion in children also produced measurable cerebral blood flow alterations despite negative conventional MRI and without clear spectroscopy evidence of neuronal metabolite depletion, indicating that vascular dysregulation may be one of the more sensitive detectable abnormalities after mild injury (68). Acute adolescent concussion was associated with altered vasoreactivity and autoregulation, and a change in vasoreactivity over 8 weeks was related to aerobic exercise volume (22). Several years after concussion, the youth still showed regional hypo- and hyperperfusion relative to orthopaedic controls (23). In adolescent athletes exposed to repetitive head impacts, transcranial Doppler measures of cerebrovascular reactivity and neurovascular coupling changed over a single season, again suggesting that vascular-fluid regulation is dynamic even below the threshold of catastrophic injury (44). Dynamic cerebral autoregulation was impaired in adolescents early after concussion and improved in some individuals after symptom resolution, although recovery trajectories were heterogeneous (69). These data broaden the relevance of CSF-dynamic thinking beyond intensive care and into the ambulatory concussion population.

Chronic or delayed physiological abnormality is also evident in broader TBI populations. In adults with persistent post-traumatic symptoms, the dynamic cerebral autoregulation phase remained impaired at least 6 months after injury and correlated with poorer cognitive performance (70). Although not paediatric, this mixed-age evidence is relevant because it shows that flow-regulatory abnormalities can outlast the acute lesion and remain clinically meaningful. It strengthens the plausibility of a model in which PTH is the delayed consequence of unresolved physiological dysregulation rather than simply of acute obstruction.

### Imaging markers of altered cerebrospinal fluid dynamics

Several imaging markers are relevant to altered CSF regulation after trauma, although none is sufficient in isolation. Glymphatic-associated imaging produced one of the most interesting, yet most internally inconsistent, sets of findings. In moderate-to-severe TBI with diffuse axonal injury, the ALPS index was significantly lower than in TBI without DAI and correlated negatively with DAI grade (24). By contrast, in retrospective mild TBI cohorts, ALPS indices were reported as higher than in controls, including in MRI-negative cases, and in young boxers with sports-related concussion, DTI-ALPS values were also higher and correlated with poorer cognitive results (25, 43). Read conservatively, these studies suggest that glymphatic or perivascular imaging abnormalities are present after head injury but may be directionally heterogeneous according to injury type, timing, or measurement context.

Perivascular spaces are another potential marker. Inglese et al. reported that patients with mild TBI had significantly more high-convexity dilated Virchow-Robin spaces than controls, and that this abnormality did not simply track with age or global CSF volume (71). Although not paediatric, this finding is conceptually relevant to trauma-related disturbances in clearance and supports the broader notion that perivascular morphology may register injury-related alterations in fluid exchange.

Extra-axial spaces also matter in infancy. In accidental head trauma, enlarged subarachnoid spaces were associated with larger head circumference percentile and with subarachnoid or subpial haemorrhage, but not with subdural haemorrhage or epidural haemorrhage (72). In a larger non-trauma cohort of children with enlarged subarachnoid spaces, the frequency of subdural haemorrhage was low, complicating assumptions that widened extra-axial spaces necessarily predispose to subdural bleeding (73). These studies are important diagnostically because post-traumatic ventriculomegaly or extra-axial fluid enlargement may coexist with developmental enlargement of the subarachnoid space.

Normative studies provide the reference framework for interpreting these markers. Ultrasound work showed that subarachnoid space width varies with age in normal infants and children (74). CT and MRI studies established age- and sex-related normal values for optic nerve sheath diameter, optic nerve diameter, and eyeball transverse diameter in children (37–39). These measures are not direct CSF flow metrics, but in clinical practise, they are often used as accessible surrogates of ICP or subarachnoid pressure transmission, and their normal developmental variation is therefore highly relevant to post-traumatic assessment (37–39).

### Synthesis of post-traumatic cerebrospinal fluid dysfunction

Across the acute, subacute, and chronic studies, a coherent picture emerges. Immediately after injury, some children develop low CBF, others hyperaemia, and many exhibit impaired autoregulation, low compliance reserve, or oxygenation deficits despite apparently adequate mean pressures (18, 19, 42, 46–48, 54). Over the following weeks and months, cerebrovascular reactivity, CBF, and glymphatic-associated imaging may remain abnormal in the absence of frank hydrocephalus (20–25). Only later, in a subset, does this evolving physiological disturbance become recognisable as ventriculomegaly or symptomatic CSF outflow abnormality (5).

This progression supports a hypothesis-generating framework in which post-traumatic hydrocephalus is considered as one possible late manifestation of dynamic CSF dysregulation. The difficulty is that the paediatric literature documents many elements of this pathway separately, but rarely follows the same patients from acute physiological disturbance to delayed hydrocephalus. That gap remains a major weakness of the evidence base (5, 12, 20).

## Post-traumatic hydrocephalus in children

### Definitions and classification

For the purposes of this review, several related but distinct terms are used. Post-traumatic hydrocephalus refers to ventricular enlargement after traumatic brain injury that is associated with clinical deterioration, raised intracranial pressure, impaired CSF outflow or reserve, or responsiveness to CSF diversion. This is distinguished from post-traumatic ventriculomegaly, which describes radiological ventricular enlargement after trauma without necessarily implying raised pressure, impaired CSF absorption, or shunt-responsive hydrocephalus. Ex vacuo ventriculomegaly refers to ventricular enlargement secondary to parenchymal volume loss, encephalomalacia, or cerebral atrophy, rather than a primary disorder of CSF circulation. Communicating hydrocephalus is used to describe hydrocephalus without macroscopic obstruction within the ventricular system, usually reflecting impaired CSF absorption, altered intracranial compliance, or distributed outflow dysfunction. By contrast, non-communicating hydrocephalus refers to hydrocephalus caused by obstruction within the ventricular system or at a defined CSF pathway, such as aqueductal stenosis or fourth ventricular outlet obstruction.

Hydrocephalus is commonly framed in terms of communicating versus non-communicating physiology, post-traumatic ventriculomegaly, or broader disturbances of CSF dynamics (5, 11, 30). This is not merely semantic. If PTH is conceptualised narrowly as ventricular enlargement requiring shunting, many earlier stages of clinically relevant dysregulation are excluded. If it is conceptualised too broadly, it becomes difficult to distinguish from ex-vacuo change, benign enlargement of subarachnoid spaces, or compensated post-traumatic compliance abnormality.

The most direct PTH study, by Lalou et al., focused on non-acute post-traumatic ventriculomegaly and used infusion testing to explore Rout, pulse amplitude, RAP, and elasticity (5). Its importance lies partly in what it compared PTH against. By benchmarking post-traumatic ventriculomegaly against shunt-responsive idiopathic normal pressure hydrocephalus, Lalou et al. showed that post-traumatic cases had lower AMP and dAMP than iNPH yet still displayed reduced compliance and, in shunted probable PTH, higher Rout (5). This suggests that post-traumatic hydrocephalus is not simply a younger analogue of iNPH and that its diagnostic signature may involve a different balance between outflow resistance, compliance failure, and radiological change.

That design is noteworthy because it effectively treats post-traumatic hydrocephalus as a physiological diagnosis within a radiological phenotype. MRI flow studies of communicating hydrocephalus in children further suggest that hydrocephalus is not a single flow state: infant and older-child patterns differed in the direction and magnitude of aqueductal net flow (11). The literature, therefore, supports classification by dynamics as much as by anatomy.

### Incidence and risk factors

Direct incidence data and validated paediatric risk models for post-traumatic hydrocephalus remain limited. Younger age was associated with worse perfusion profiles and poorer outcomes after severe TBI in several cohorts (46, 47). Delayed intracranial hypertension was associated with oedema on the initial CT scan (50). Impaired autoregulation predicted poor outcome in severe paediatric TBI and appeared particularly severe in inflicted injury (19, 45). In STARSHIP, greater duration and magnitude of reactivity disturbance, lower tolerated CPP relative to individual autoregulatory thresholds, and worse compliance metrics were all associated with poor outcome (12–15). These are not direct PTH risk factors, but they describe physiological states that plausibly predispose to later failure of CSF compensation.

The only direct post-traumatic ventriculomegaly study adds one further clue: amongst patients selected for shunting, Rout was significantly higher than in unshunted patients (5). That does not tell us who develops PTH, but it does indicate that when post-traumatic ventriculomegaly becomes clinically important, altered outflow resistance is part of the phenotype.

### Pathophysiological mechanisms

Several interacting mechanisms of PTH are biologically plausible, but few have been directly demonstrated in paediatric longitudinal studies. The strongest direct evidence concerns reduced compliance and altered outflow resistance. In non-acute post-traumatic ventriculomegaly, Lalou et al. found lower pulse amplitude and dAMP than in shunt-responsive idiopathic normal pressure hydrocephalus, higher-than-normal elasticity in all groups, and higher Rout in probable PTH cases selected for shunting (5). Their conclusion was that compliance appears reduced in PTH and that physiological testing may detect clinically relevant hydrocephalus even when imaging descriptions are inconsistent (5).

Hydrocephalus-related MRI flow studies suggest that abnormal flow can be present, but that simple flow thresholds are not diagnostically robust. In children with communicating hydrocephalus, older patients had seven-fold higher aqueductal net flow than controls, while infants showed altered flow direction with preserved magnitude (11). In adults or mixed-age communicating hydrocephalus, aqueductal stroke volume and peak velocities were higher than in healthy subjects, but the large overlap between patients and controls limited individual diagnostic use (75, 76). These data imply that pathological CSF dynamics can be measurable, but not by a single universal metric.

The broader trauma physiology literature suggests several mechanisms by which such abnormalities could develop. Early oedema, increased cerebral blood volume, impaired autoregulation, and repeated ICP insults all alter the pressure–volume environment of the injured brain (15, 18, 19, 50, 51). Reduced compliance of the CSF space and altered vascular closing pressures suggest that the injured brain loses buffering capacity and may transmit pulsatile loads abnormally (29, 42). Perivascular or glymphatic alterations after TBI, whether reflected by lower ALPS in DAI or altered ALPS in mild injury, provide an additional potential mechanism for impaired solute and fluid clearance (24, 25, 43). None of these findings alone proves causation for PTH, but together they support a model in which post-traumatic hydrocephalus represents the cumulative failure of several regulatory subsystems.

### Clinical presentation and diagnostic challenges

Several studies repeatedly warn against relying solely on morphology. Lalou et al. noted that mild or moderate hydrocephalus, ex-vacuo ventriculomegaly, and encephalomalacia were inconsistently reported in their post-traumatic ventriculomegaly patients, even when infusion testing later identified differences relevant to shunt selection (5). Earlier phase-contrast studies also showed marked overlap in aqueductal flow measures between normal and dilated ventricles or between communicating hydrocephalus and controls (34, 35, 76). In short, ventricular size, aqueductal flow, and symptom burden may all dissociate. The diagnostic task is therefore not simply to label a picture as “hydrocephalus,” but to decide whether the measured anatomy is behaving like a decompensated fluid system.

Infancy introduces further diagnostic complexity. Enlarged subarachnoid spaces can be physiological or developmental, age-dependent, and associated with a large head circumference without implying traumatic hydrocephalus (72–74). Theoretical work has proposed that benign external hydrocephalus could increase susceptibility to extra-axial bleeding after minor trauma, but a clinical accidental-trauma series did not find an association between enlarged SAS and subdural haemorrhage (72, 77). These studies matter because enlarged extra-axial spaces, head growth, and post-traumatic collections can obscure the interpretation of evolving ventricular or subarachnoid enlargement after injury.

The clinical challenge, then, is that PTH may present as a dynamic mismatch rather than a dramatic anatomy. This helps explain why imaging-alone frameworks often struggle in the post-traumatic setting. Ventricles may enlarge because of atrophy, altered pulsatility, reduced compliance, or true outflow resistance; extra-axial spaces may be developmentally large; and acute traumatic collections may contain mixtures of blood and CSF rather than representing a single chronic process (5, 72, 73, 78). A child may have deteriorating reserve, abnormal pulsatility, or rising Rout before ventricular size becomes unequivocal. Conversely, a child with enlarged ventricles may not have true hydrocephalus. The literature reviewed here therefore supports a physiology-first diagnostic mindset: suspected PTH should be assessed by integrating clinical course, developmental context, imaging, and, where possible, direct dynamic testing (5, 11, 34, 76).

## Assessment of cerebrospinal fluid dynamics in suspected post-traumatic hydrocephalus

### Clinical assessment

Direct clinical assessment remains necessary because imaging and physiology each capture only part of the problem. In infants and very young children, head growth and extra-axial space size are central to interpretation. Ultrasound studies have shown that the subarachnoid space width varies with age in normal children, while CT studies of accidental trauma have linked enlarged extra-axial spaces to higher head circumference percentile (72, 74). MRI volumetric work further demonstrated an age-dependent brain-to-CSF ratio across childhood (9). These normative data support the use of trajectories rather than single measurements when assessing possible CSF dysregulation.

There is no unified clinical scoring system for suspected paediatric PTH. What is suggestive is that physiology can sharpen bedside suspicion. In severe paediatric TBI, non-invasive ONSD correlated better with disease severity and long-term outcome than pulsatility index–derived estimates, although ONSD changed too slowly to be a useful short-interval follow-up tool (79). That pattern is clinically plausible for suspected PTH, where the question is often whether a child is drifting into sustained pressure or reserve failure rather than whether minute-to-minute ICP is changing.

### Radiological assessment

CT and conventional MRI remain necessary for identifying ventriculomegaly, extra-axial collections, mass lesions, and parenchymal loss, but advanced imaging can enrich interpretation. Phase-contrast MRI studies provide normative aqueductal values in healthy children and show how hydrocephalic patterns may deviate in direction, magnitude, velocity, or pulsatility (6, 11, 32, 33). Yet these same studies also demonstrate considerable inter-individual variability and overlap, limiting their use as stand-alone decision tools (34, 35, 76).

Optic nerve sheath measures may help in selected situations but require a paediatric normative context. CT and MRI studies established that ONSD varies with age and, in some series, sex, while ONSD/ETD ratios may be more stable (37–39). In moderate-to-severe paediatric TBI, ONSD-based non-invasive ICP estimates correlated with severity and long-term outcome better than pulsatility-index–derived estimates, but ONSD changed relatively slowly and was less useful as an acute follow-up tool (79). That distinction is relevant to suspected PTH: ONSD may reflect a sustained pressure burden better than rapid, short-term fluctuations.

Perivascular and glymphatic imaging remains promising but not yet definitive. Normative childhood ALPS values are age dependent (7). After injury, ALPS abnormalities have been observed in DAI, mild TBI, and sports-related concussion (24, 25, 43). These findings indicate biological plausibility for glymphatic assessment in PTH; however, these do not define diagnostic thresholds or longitudinal trajectories that would allow ALPS to function as a clinical test for paediatric PTH.

### Invasive monitoring and infusion studies

Where uncertainty persists, invasive dynamic assessment may be the most informative approach. This includes both continuous monitoring and provocative testing. Continuous metrics, such as PRx, pulse-shape analysis, and compliance proxies, can detect deterioration before major ICP insults are established (12, 15, 16, 18). Dynamic CPP analyses showed that fixed thresholds may not reflect individual autoregulatory needs, with lower autoregulation limits often exceeding traditional targets (14, 49, 80). Infusion studies, in contrast, interrogate outflow and reserve more directly, as in the Lalou ventriculomegaly series (5). These tools were not developed specifically for PTH, but they demonstrate that clinically meaningful physiology can be extracted from time-series monitoring rather than static measurements. The conceptual advantage of combining these methods is that one describes spontaneous system behaviour over time while the other tests the system’s response to volume stress.

The one study directly addressing post-traumatic ventriculomegaly used CSF infusion testing. Lalou et al. measured Rout, pulse amplitude, RAP, and elasticity, and showed that shunted probable PTH cases had higher Rout than unshunted cases, while imaging descriptors of hydrocephalus and atrophy were inconsistent (5). This is arguably the most important diagnostic study because it shows that physiology can discriminate clinically relevant post-traumatic ventriculomegaly where morphology alone may not (5).

At the same time, the broader hydrocephalus literature cautions against single-parameter tests. PVI, aqueductal stroke volume, and oscillatory flow measures are all informative but show substantial overlap between normal and pathological states (35, 65, 76). Thus, the best interpretation of the evidence is that suspected PTH should be assessed multimodally: clinical course, age-calibrated morphology, flow imaging, and invasive dynamic testing where clinically justified.

## Management implications of altered cerebrospinal fluid dynamics

### Surgical intervention

Direct paediatric trauma-specific comparative data on CSF diversion strategies are lacking. However, the available physiological literature supports a framework in which surgical diversion is most likely to be appropriate when symptoms, radiology, and dynamic evidence of impaired reserve or outflow converge. No specific operation can be recommended as superior on the basis of the current evidence. In other words, matching the intervention to the dominant physiology: outflow resistance and reduced reserve favour diversion, whereas mere enlargement without physiological corroboration does not automatically imply a shunt-responsive state (5, 34, 76). This suggests that when children or young adults present with post-traumatic ventricular enlargement and diagnostic uncertainty, dynamic evidence of impaired outflow and reserve may be more helpful than ventricular size alone (5).

The non-traumatic hydrocephalus studies also provide useful cautionary lessons. Aqueductal flow metrics differed at the group level between communicating hydrocephalus and controls, but the overlap between individuals was large enough that flow measures alone were not suitable for shunt screening (76). In children with normal and dilated ventricles, marked inter-individual variation limited interpretation of isolated aqueductal measurements (34). These findings argue against over-reliance on “high-flow” or “low-flow” MRI as a proxy for treatment responsiveness.

### Outcomes and complications

Because paediatric intervention literature specific to post-traumatic hydrocephalus is sparse, treatment outcomes such as shunt dependency rates, revision rates, and neurodevelopmental trajectories after trauma-specific CSF diversion are not specified. However, several studies indirectly support the importance of identifying treatable physiology before irreversible decline. Continuous reactivity and compliance abnormalities were associated with worse outcomes after severe paediatric TBI (12, 15–17). In mild TBI, altered CBF trajectories were associated with delayed recovery (21). These findings do not prove that earlier CSF diversion changes outcome, but they do show that fluid-related physiological derangement has prognostic importance.

### Towards physiology-informed decision-making

The clearest management implication is that physiology should be matched to intervention. If a child with post-traumatic ventriculomegaly has imaging enlargement but no physiological evidence of impaired outflow or reserve, immediate diversion may be inappropriate. Conversely, if imaging change is modest but dynamic studies suggest elevated Rout, impaired compliance, abnormal pulsatility, or progressive pressure dysregulation, hydrocephalus may be clinically important despite equivocal scans (5, 15, 29).

A second implication is that over-simplified pressure targets may be inadequate. The STARSHIP analyses are especially important here because they suggest that lower limits of autoregulation often sit above fixed guideline CPP floors, including in the youngest children, for a substantial proportion of monitoring time (14). KidsBrainIT similarly visualised a “safe” CPP zone rather than a single universal threshold and showed that this zone shrinks when cerebrovascular reactivity is passive, or ICP is high (80). If clinicians accept that later CSF failure may partly reflect earlier periods of under-resuscitated dysautoregulation, then acute haemodynamic management becomes part of hydrocephalus prevention, not just part of general neurocritical care.

These observations also have implications for trial design and prevention strategies. If lower limits of autoregulation vary between patients and may lie above conventional guideline thresholds, future studies of post-traumatic hydrocephalus prevention should integrate personalised haemodynamic targets rather than assume that guideline-level cerebral perfusion pressure represents physiological adequacy (12, 14, 80, 81).

A third implication is diagnostic humility. Studies consistently show overlap between normal and pathological imaging metrics, age dependence in physiological baselines, and heterogeneity in post-traumatic responses (5, 7, 8, 11, 32, 33, 76). A physiology-informed pathway should therefore seek concordance across multiple domains rather than a single decisive test.

## Discussion

The most obvious gap in the literature is the scarcity of direct paediatric post-traumatic hydrocephalus studies. This scarcity is itself clinically important because it exposes a mismatch between how often clinicians discuss post-traumatic hydrocephalus and how little paediatric dynamic evidence is actually available. The literature is rich in surrogates of disturbed cerebrospinal fluid regulation and poor outcome, but is poor in studies that verify which of those surrogates forecast later hydrocephalus, which are transient, and which are treatment-responsive (5, 12, 15, 20, 21).

This scarcity has an important methodological consequence. Much of the pathway proposed in this review must be inferred from adjacent physiological domains—autoregulation, perfusion, oxygenation, compliance, concussion physiology, and normal cerebrospinal fluid development—rather than from longitudinal paediatric hydrocephalus cohorts (5).

A second gap is the absence of standardised CSF dynamic metrics across studies. Different investigators use PRx, ARI, Mx, wPRx, PAx, RAC, CPPopt, lower limit of autoregulation, pulse-shape indices, compliance proxies, aqueductal stroke volume, ALPS indices, optic nerve sheath measures, and infusion-test parameters (5, 11, 12, 14–17, 29, 42). This diversity is intellectually productive but clinically fragmenting. Without common definitions and harmonised thresholds, it is difficult to translate the literature into a pragmatic diagnostic algorithm for suspected PTH.

A third gap is longitudinal linkage. Severe TBI studies often focus on the first hours to days after injury, while mild TBI studies focus on weeks to months after concussion (12, 18–23, 46). What is largely missing is a study design that starts with acute multimodal physiology, follows patients through subacute imaging change, and then determines who develops symptomatic ventriculomegaly, who requires diversion, and who improves spontaneously. That is exactly the study structure needed to validate the thesis that PTH is a delayed manifestation of early dynamic dysregulation.

A fourth gap concerns developmental calibration. Normative paediatric studies show strong age effects in brain-CSF ratios, aqueductal flow direction, glymphatic indices, fontanelle pressure characteristics, and compliance-related measures (7, 9–11, 40). Future trauma studies must therefore incorporate age-stratified reference frameworks, especially in infancy and early childhood, rather than applying pooled paediatric thresholds.

There is a need to integrate imaging, physiology, and outcome in a way that is clinically tractable. The ideal future PTH study in children would combine age-calibrated volumetry, phase-contrast flow, perivascular or glymphatic markers, continuous autoregulation and compliance monitoring, and prospective neurodevelopmental follow-up. It would also test whether a physiology-guided intervention improves outcomes compared with morphology-guided care. The literature strongly supports such a programme but has not yet reported its results (5–7, 12, 14, 20).

A number of interpretive points emerge from the evidence base. First, the literature is much more consistent about the existence of post-traumatic physiological dysregulation than about the exact metric that best captures it. Whether the monitored variable is PRx, lower limit of autoregulation, CPP dose, pulse-shape index, compliance proxy, PbtO2, ALPS index, or aqueductal flow, the direction of travel is similar: children with worse trajectories tend to show poorer reserve, poorer coupling, or greater departure from developmental norms (12–15, 20, 21, 24, 29, 42). The implication is that the field may be measuring different windows onto the same underlying disorder.

Second, the studies consistently undermine reductionist interpretations. High CPP is not necessarily harmful in all children, low CPP is not always captured by fixed thresholds, normal-sized ventricles do not exclude major physiological disturbance, and enlarged ventricles do not prove shunt-responsive hydrocephalus (5, 14, 76, 80, 82). Likewise, ALPS abnormalities after TBI are not directionally uniform across injury phenotypes (24, 25, 43). These apparent inconsistencies are not simply noise. They suggest that paediatric CSF dysregulation after trauma is context dependent, varying with age, severity, injury mechanism, timing, and the specific subsystem being measured.

Third, the developmental literature is not a background luxury but a prerequisite for correct diagnosis. The normal child has age-dependent changes in brain-to-CSF ratio, aqueductal flow direction, spinal and ventricular pulsatility, compliance-related waveform measures, fontanelle pressure behaviour, and optic nerve sheath dimensions (6, 8–11, 37–39). Without those baselines, paediatric post-traumatic physiology can easily be overcalled or undercalled. This is especially true in infancy, where developmental enlargement of extra-axial spaces and age-specific flow patterns can mimic or mask pathological CSF disturbance (11, 72–74).

There is also a methodological lesson. The most clinically useful literature comes from high-resolution or repeated-measures physiology rather than one-off snapshots. STARSHIP, KidsBrainIT, longitudinal concussion perfusion studies, and infusion testing all derive part of their value from observing systems over time or under stress rather than at a single static moment (5, 12, 14, 15, 21, 80). That is probably the correct direction of travel for PTH research. Hydrocephalus after trauma is likely to be a time-dependent systems failure, and time-dependent systems failures are unlikely to be captured by single images or thresholds alone.

A final point concerns diagnostic language. In day-to-day neurosurgical practise, the label “hydrocephalus” often functions as a binary decision term because it is tied to whether a child needs CSF diversion. Binary framing may be clinically convenient but physiologically crude. Between clearly compensated normality and clearly shunt-responsive hydrocephalus lies a large intermediate zone of disturbed fluid regulation, in which autoregulation, compliance, pulsatility, oxygenation, and clearance are abnormal but anatomy remains ambiguous (5, 12, 29, 42). Recognising that the intermediate zone may be the key advance needed to diagnose paediatric PTH earlier and more accurately.

### Limitations

This review has several limitations. First, direct paediatric evidence linking acute CSF-related physiological disturbance to later post-traumatic hydrocephalus is sparse. Second, several mechanistic elements of the proposed model are derived from adjacent paediatric TBI physiology, normative developmental CSF studies, or adult/mixed-population studies rather than paediatric PTH cohorts. Third, the reviewed studies use heterogeneous physiological metrics and thresholds, limiting direct comparison. Fourth, the proposed physiology-informed framework should be regarded as hypothesis-generating and should not be interpreted as a validated diagnostic or treatment algorithm.

## Conclusion

This review supports a cautious, hypothesis-generating reframing of paediatric post-traumatic hydrocephalus as a condition that may involve dynamic CSF dysregulation in addition to structural obstruction, impaired absorption, and ex vacuo ventricular enlargement. The strongest direct evidence comes from post-traumatic ventriculomegaly studies using CSF infusion testing, whereas much of the broader argument is inferred from paediatric TBI physiology, normative developmental CSF studies, and adult or mixed-population mechanistic work. Current evidence supports multimodal assessment and developmental interpretation of suspected PTH, but does not yet validate a specific diagnostic algorithm or treatment pathway. Future longitudinal paediatric studies should link acute autoregulation, compliance, perfusion, pulsatility, CSF outflow, and glymphatic/perivascular markers with delayed ventriculomegaly, treatment response, and neurodevelopmental outcome.
